# Abnormal White Matter Integrity in Adolescents with Internet Addiction Disorder: A Tract-Based Spatial Statistics Study

**DOI:** 10.1371/journal.pone.0030253

**Published:** 2012-01-11

**Authors:** Fuchun Lin, Yan Zhou, Yasong Du, Lindi Qin, Zhimin Zhao, Jianrong Xu, Hao Lei

**Affiliations:** 1 State Key Laboratory of Magnetic Resonance and Atomic and Molecular Physics, Wuhan Center for Magnetic Resonance, Wuhan Institute of Physics and Mathematics, Chinese Academy of Sciences, Wuhan, People's Republic of China; 2 Department of Radiology, RenJi Hospital, Jiao Tong University Medical School, Shanghai, People's Republic of China; 3 Department of Child and Adolescent Psychiatry, Shanghai Mental Health Center, Jiao Tong University, Shanghai, People's Republic of China; Université de Montréal, Canada

## Abstract

**Background:**

Internet addiction disorder (IAD) is currently becoming a serious mental health issue around the globe. Previous studies regarding IAD were mainly focused on associated psychological examinations. However, there are few studies on brain structure and function about IAD. In this study, we used diffusion tensor imaging (DTI) to investigate white matter integrity in adolescents with IAD.

**Methodology/Principal Findings:**

Seventeen IAD subjects and sixteen healthy controls without IAD participated in this study. Whole brain voxel-wise analysis of fractional anisotropy (FA) was performed by tract-based spatial statistics (TBSS) to localize abnormal white matter regions between groups. TBSS demonstrated that IAD had significantly lower FA than controls throughout the brain, including the orbito-frontal white matter, corpus callosum, cingulum, inferior fronto-occipital fasciculus, and corona radiation, internal and external capsules, while exhibiting no areas of higher FA. Volume-of-interest (VOI) analysis was used to detect changes of diffusivity indices in the regions showing FA abnormalities. In most VOIs, FA reductions were caused by an increase in radial diffusivity while no changes in axial diffusivity. Correlation analysis was performed to assess the relationship between FA and behavioral measures within the IAD group. Significantly negative correlations were found between FA values in the left genu of the corpus callosum and the Screen for Child Anxiety Related Emotional Disorders, and between FA values in the left external capsule and the Young's Internet addiction scale.

**Conclusions:**

Our findings suggest that IAD demonstrated widespread reductions of FA in major white matter pathways and such abnormal white matter structure may be linked to some behavioral impairments. In addition, white matter integrity may serve as a potential new treatment target and FA may be as a qualified biomarker to understand the underlying neural mechanisms of injury or to assess the effectiveness of specific early interventions in IAD.

## Introduction

Internet addiction disorder (IAD), also called problematic or pathological Internet use, is characterized by an individual's inability to control his or her use of the Internet, which may eventually result in marked distress and functional impairments of general life such as academic performance, social interaction, occupational interest and behavioral problems [1]. The description regarding IAD is based on the definition for substance dependence or pathological gambling, which shares properties of substance dependence like preoccupation, mood modification, tolerance, withdrawal, distress and functional impairments [2], [3]. With the soaring number of Internet users, the problem of IAD has currently attracted considerable attention from psychiatrists, educators and the public; therefore IAD is becoming a serious mental health issue around the world [4], [5], [6].

Current studies about IAD have focused on case summaries, behavioral components, negative consequences in daily life, along with clinical diagnosis, epidemiology, associated psychosocial factors, symptom management, psychiatric comorbidity and treatment outcome [7], [8], [9], [10], [11]. These studies are mainly based on psychological self-reported questionnaires and consistently reported that heavy internet overuse may exert potential effects on individuals' psychological problems and cognitive impairments.

To date, only few neuroimaging studies had been performed to investigate brain structural and functional changes associated with IAD. A previous voxel-based morphometry (VBM) study reported decreased gray matter density in the left anterior cingulate cortex, posterior cingulate cortex, insula and lingual gyrus of IAD adolescents [12]. Yuan and colleagues found that IAD subjects had multiple structural changes in the brain, and such changes correlated significantly with the duration of Internet addiction [13]. One resting-state functional magnetic resonance imaging (fMRI) study demonstrated that IAD college students has increased regional homogeneity in several brain regions including cerebellum, brainstem, limbic lobe, frontal lobe and apical lobe [14]. Two task-related fMRI studies of individuals with online game addiction indicated that cue-induced activation in response to Internet video game stimuli is similar to that observed during cue presentation in people with substance dependence or pathologic gambling [15], [16]. Dong et al. [17] reported that IAD students had lower activation in the conflict detection stage, and showed less efficiency in information processing and lower impulse control than normal controls by recording event-related brain potentials during a Go/No-Go task. Additionally, a positron emission tomography (PET) study found that Internet game overuse shares psychological and neural mechanisms with other types of impulse control disorders and substance/non-substance-related addiction [18]. Taken together, these findings indicate that IAD subjects are associated with structural and functional changes in brain regions involving in emotional processing, executive attention, decision making and cognitive control.

We hypothesize that IAD subjects are also associated with impairments of white matter fibers connecting these regions and such changes can be detected by diffusion tensor imaging (DTI), a non-invasive MRI technique with capable of providing a quantitative measure of white matter damage [19]. DTI is sensitive to water diffusion characteristics and has been developed as a tool for investigating the local properties of brain white matter [20]. Four frequently used quantitative diffusion parameters can be derived from DTI data: 1) fractional anisotropy (FA), reflecting the directionality of water diffusion and coherence of white matter fiber tracts; 2) mean diffusivity (MD), quantifying the overall magnitude of water diffusion; 3) axial diffusivity (Da) measuring the magnitude of diffusivity along the principle diffusion direction; and 4) radial diffusivity (Dr) reflecting the magnitude of diffusivity perpendicular to the principle diffusion direction [21], [22]. These measures are related to the microstructural organization of white matter and used to infer structural characteristics of the local tissue environment.

In this study, we used DTI to investigate the white matter integrity in adolescents with IAD. An observer-independent tract-based spatial statistics (TBSS) analysis method was used to analyze the DTI data. This method retains the strengths of voxel-based analysis while addressing some of its drawbacks, such as aligning images from multiple subjects and the arbitrariness of the choice of spatial smoothing [23]. The aims of the study are 1) to investigate differences in the topographic distribution of white matter integrity between adolescents with IAD and healthy controls without IAD, making no *a priori* assumptions about the location of possible abnormalities, and 2) to determine whether there was any relationship between white matter integrity and neurophysiological measures in IAD subjects.

## Materials and Methods

### Subjects

Eighteen adolescents with IAD were recruited from the Department of Child and Adolescent Psychiatry, Shanghai Mental Health Center, all of whom met the modified Young's diagnostic questionnaire for internet addiction criteria by Beard and Wolf [2]. Eighteen age, gender and yeas of education matched normal subjects without IAD were selected as controls. All subjects were right-handed as evaluated by a questionnaire according to the Edinburgh handedness inventory [24]. The structural MRI data from these subjects had been used in our previous VBM study [12]. For this study, the imaging data from two controls and one IAD subject had to be discarded because of large motion artifacts. As a result, a total of sixteen controls (age range: 15–24) and seventeen IAD subjects (age range: 14–24) were included. The demographic information of the subjects included is listed in Table 1.

**Table 1:** Demographic and behavioral characteristics of the included participants.

	CON (n = 16)	IAD (n = 17)	*p* value
	(Mean±SD)	(Mean±SD)	
Age	17.78±2.46	17.01±2.50	0.38
Gender (M/F)	14/2	15/2	0.95
Education (years)	11.50±2.99	10.47±2.40	0.28
Young's Internet Addiction Scale (YIAS)	37.00±10.64	64.71±12.58	**<0.0001**
Time Management Disposition Scale (TMDS)	123.60±20.17	124.00±22.80	0.96
Strength and Difficulties Questionnaire (SDQ)	16.40±3.87	21.76±3.46	**<0.001**
Barratt Impulsiveness Scale-11 (BIS)	67.20±7.83	69.82±12.34	0.49
The Screen for Child Anxiety Related Emotional Disorders (SCARED)	24.71±6.16	38.59±9.90	**<0.0001**
Family Assessment Device (FAD)	117.73±10.89	129.12±13.93	**0.016**

Abbreviation. CON: controls; IAD: Internet addiction disorder; SD: standard deviation.

Two-sample *t* test was used for group comparisons but chi-square was used for gender comparison.

The study was approved by the Ethics Committee of RenJi Hospital of Shanghai Jiao Tong University Medical School. The participants and their parents/legal guardians were informed of the aims of our study before MRI examinations. Full written informed consent was obtained from the parents/guardians of each participant.

### Inclusion and Exclusion Criteria

All subjects underwent a simple physical examination including blood pressure and heart rate measurements, and were interviewed by a psychiatrist regarding their medical history on nervous, motion, digestive, respiratory, circulation, endocrine, urinary and reproductive systems. They were then screened for psychiatric disorders with the Mini International Neuropsychiatric Interview for Children and Adolescents (MINI-KID) [25]. The exclusion criteria included a history of substance abuse or dependence; a history of major psychiatric disorders, such as schizophrenia, depression, anxiety disorder, psychotic episodes, or hospitalization for psychiatric disorders. The IAD subjects were not treated with any medications. However, a small number of IAD subjects received psychotherapy.

The diagnostic standard for IAD was adapted from the modified Young's Diagnostic Questionnaire for Internet Addiction criteria by Beard and Wolf [2]. The criteria consisting of eight ‘yes’ or ‘no’ items was translated into Chinese. It includes the following questions: (1) Do you feel preoccupied with the Internet (i.e., think about previous online activity or anticipate next online session)? (2) Do you feel the need to use the Internet with increasing amounts of time in order to achieve satisfaction? (3) Have you repeatedly made unsuccessful efforts to control, cut back or stop Internet use? (4) Do you feel restless, moody, depressed, or irritable when attempting to cut down or stop Internet use? (5) Do you stay online longer than originally intended? (6) Have you jeopardized or risked the loss of a significant relationship, job, educational or career opportunity because of the Internet? (7) Have you lied to family members, a therapist or others to conceal the extent of involvement with the Internet? (8) Do you use the Internet as a way of escaping from problems or of relieving a distressed mood (e.g., feelings of helplessness, guilt, anxiety, and depression)? Participants who answered ‘yes’ to items 1 through 5 and at least any one of the remaining three items were classified as suffering from IAD.

### Behavioral assessments

Six questionnaires were used to assess the participants' behavioral features, namely the Young's Internet Addiction Scale (YIAS) [26], Time Management Disposition Scale (TMDS) [27], Strengths and Difficulties Questionnaire (SDQ) [28], Barratt Impulsiveness Scale-11 (BIS) [29], the Screen for Child Anxiety Related Emotional Disorders (SCARED) [30] and Family Assessment Device (FAD) [31]. All questionnaires were initially constructed in English and translated into Chinese.

### Image acquisition

Diffusion tensor imaging was performed on a 3.0-Tesla Phillips Achieva medical scanner. A single-shot echo planar diffusion weighted imaging with alignment of the anterior-posterior commissures plane was done according to the following parameters: repetition time = 8,044 ms; echo time = 68 ms; SENSE factor = 2; acquisition matrix = 128×128 zero-filled to 256×256; field of view = 256×256 mm^2^; slice thickness = 4 mm without gap. A total of 34 sections covered the whole brain including the cerebellum. The diffusion sensitizing gradients were applied along 15 non-collinear gradient encoding directions with b = 800 s/mm^2^. One additional image without diffusion gradients (b = 0 s/mm^2^) was also acquired. To enhance signal to noise ratio, imaging was repeated three times.

### Data preprocessing

All DTI data were preprocessed by the FMRIB's Diffusion Toolbox (FDT) within FMRIB's Software Library (FSL; http://www.fmrib.ox.ac.uk/fsl). First, the diffusion-weighted volumes were aligned to its corresponding non-diffusion-weighted (b_0_) image with an affine transformation to minimize image distortion from eddy currents and to reduce simple head motion. Then, non-brain tissue and background noise were removed from b_0_ image using the Brain Extraction Tool. After these steps, the diffusion tensor for each voxel was estimated by the multivariate linear fitting algorithm, and the tensor matrix was diagonalized to obtain its three pairs of eigenvalues (λ_1_, λ_2_, λ_3_) and eigenvectors. And then voxelwise values of FA, MD, Da (Da = λ_1_) and Dr (Dr = (λ_2_+λ_3_)/2) were calculated.

### TBSS analysis

Whole brain analysis of FA images was performed by using TBSS [23], which was implemented in FSL. In brief, FA maps of all subjects were first realigned to a common target and then the aligned FA volumes were normalized to a 1×1×1 mm^3^ Montreal Neurological Institute (MNI152) standard space via the FMRIB58_FA template. Thereafter, the registered FA images were averaged to generate a cross-subject mean FA image, and then the mean FA image was applied to create a mean FA skeleton which represents the main fiber tracks and the center of all fiber tracts common to the group. The mean FA skeleton was further thresholded by a FA value of 0.2 to exclude peripheral tracts where there was significant inter-subject variability and/or partial volume effects with grey matter. Following the thresholding of the mean FA skeleton, the aligned FA data of each participant was projected onto the mean skeleton to create a skeletonized FA map, by searching the area around the skeleton in the direction perpendicular to each tract, and finding the highest local FA value, and then assigning this value to the corresponding skeletal structure.

To identify FA differences between IAD subjects and normal controls, the skeletonized FA data were fed into the voxel-wise statistics analysis which is based on non-parametric approach utilizing permutation test theory. The testing was performed by the FSL randomise program, which uses 5000 random permutations. Two contrasts were estimated: IAD subjects greater than controls and controls greater than IAD subjects. Age was entered into the analysis as a covariate to ensure that any observed difference of FA between groups was independent of age-related changes. Threshold-free cluster enhancement (TFCE) [32], an alternative to conventional cluster-based thresholding which is normally compromised by the arbitrary definition of the cluster forming threshold, was used to obtain the significant differences between two groups at *p*<0.01, after accounting for multiple comparisons by controlling for family-wise error (FWE) rate. From the results of voxel-wise group comparisons, the skeletal regions showing significant inter-group differences were located and labeled anatomically by mapping the FWE-corrected statistical map of *p*<0.01 to the Johns Hopkins University (JHU)-ICBM-DTI-81 white matter (WM) labels atlas and JHU-WM Tractography Atlas in MNI space.

### Volume-of-interest analysis of diffusion indices

In order to explore the microstructural mechanisms of the observed FA changes, volume-of-interest (VOI) analysis was performed to investigate changes of diffusivity indices (Da, Dr and MD) in the regions showing FA abnormalities. To do so, the VOI masks were first extracted based on the clusters showing significant inter-group FA differences. These VOIs masks were then back- projected to the original images of each subject, and the mean values of the diffusion indices within the VOIs were calculated. After confirming normal distribution of the data by a one-sample Kolmogorov-Smirnov test, one-way analysis of covariance (ANCOVA) with group as the independent variable and diffusion indices as the dependent variables was performed, controlling for age of subjects. A statistical significance level of *p*<0.05 (Bonferroni correction for multiple comparisons) was used.

Pearson correlation analyses were used to test the correlations between FA changes within the VOIs and behavioral measures. A *p*<0.05 (uncorrected) was considered statistically significant. Step-wise multiple regression analyses with averaged FA values in VOIs as dependent variable and age, education, gender, YIAS, SDQ, SCARED, FAD, TMDS and BIS as independent variables was performed to check whether the lower FA found in the VOIs could be predicted by the scores from behavioral tests.

## Results

### Demographic and behavioral measures

Table 1 lists the demographic and behavioral measures for IAD and control subjects. There were no significant differences in the distributions of age, gender and years of education between the two groups. The IAD subjects showed higher YIAS (*p*<0.0001), SDQ (*p*<0.001), SCARED (*p*<0.0001) and FAD (*p* = 0.016) scores than the controls. No differences in TMDS and BIS scores were found between the groups.

### TBSS results

**Figure 1:**
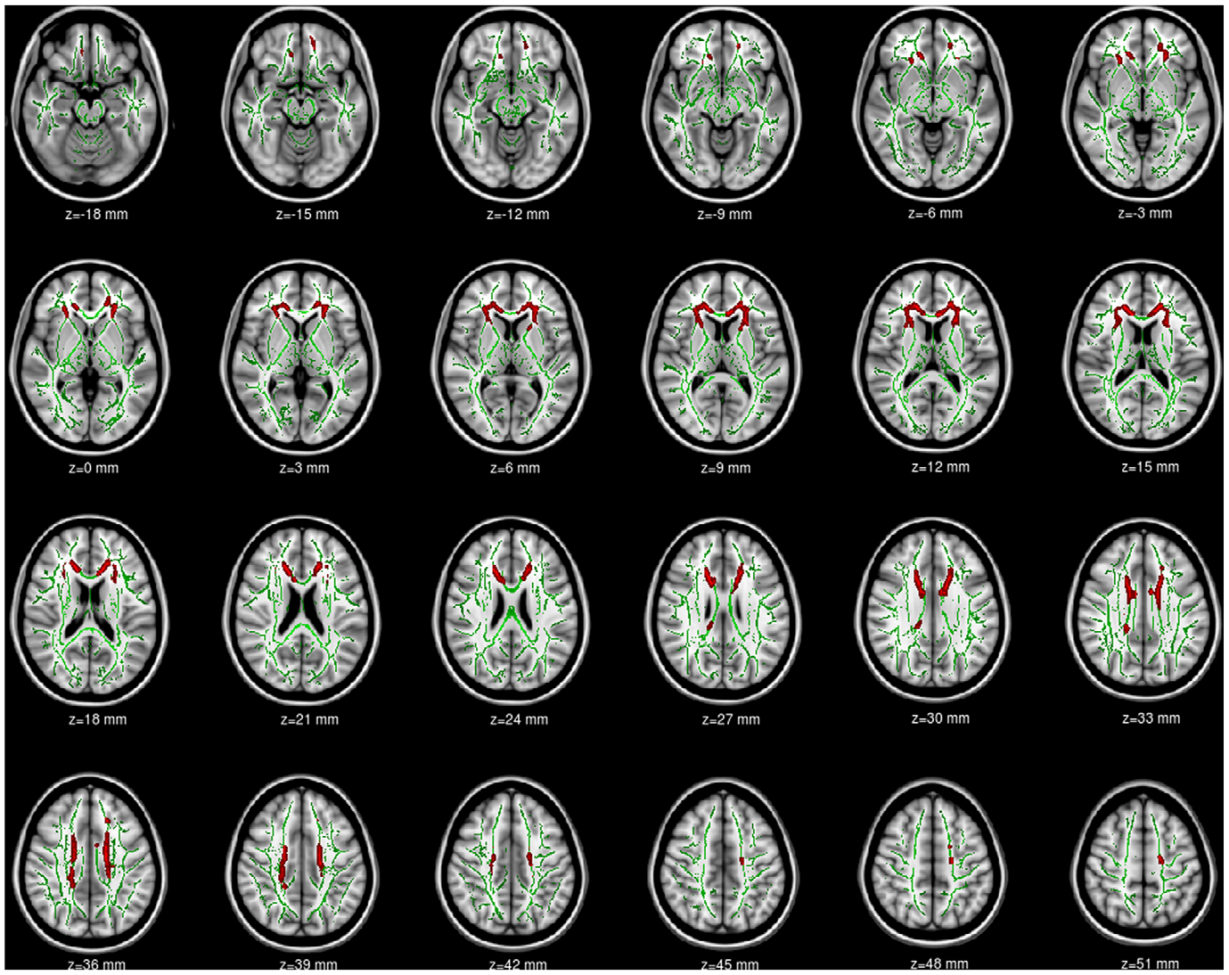
TBSS analysis of fractional anisotropy (FA) volumes. Areas in red are regions where FA was significantly lower (*p*<0.01, corrected by TFCE) in adolescents with Internet addiction disorder (IAD) relative to normal controls without IAD. To aid visualization, regions showing reduced FA (red) are thickened using the tbss_fill script implemented in FSL. Results are shown overlaid on the MNI152-T1 template and the mean FA skeleton (green). The left side of the image corresponds to the right hemisphere of the brain.

A value of 0.2 was used to threshold the mean FA skeleton volume such that a total of 131962 voxels were entered into voxel-wise TBSS analysis. The spatial distribution of the brain regions showing reduced FA in the IAD group is presented in Fig. 1 and Table 2. Compared to the control subjects, IAD subjects had significantly reduced FA (*p*<0.01; TFCE-corrected) in bilateral orbito-frontal white matter, corpus callosum, association fibers with the involvement of bilateral inferior front-occipital fasciculus and the bilateral anterior cingulum, projection fibers consisting of the bilateral anterior, superior, and posterior corona radiation, bilateral anterior limb of the internal capsule, bilateral external capsule, and left precentral gyrus. There were no white matter regions where the controls had significantly lower FA values compared with the IAD participants.

**Table 2:** Neuroanatomical regions with reduced FA in adolescents with Internet addiction disorder relative to normal controls. (*p*<0.01, TFCE corrected).

	Anatomic region	Hemisphere	MNI coordinates (mm)			*p* value(minimum)	Cluster size(mm^3^)
			X	Y	Z		
Frontal Lobe	Orbital frontal WM	R	8	40	−20	0.008	86
	Orbital frontal WM	L	−13	41	−16	0.007	119
Commissural fiber	Genu of corpus callosum	R	14	28	15	0.002	288
	Genu of corpus callosum	L	−15	31	15	0.004	241
	Body of corpus callosum	R	14	15	26	0.003	368
	Body of corpus callosum	L	−14	16	27	0.004	322
	Splenium	R	19	−34	32	0.008	81
Associate fiber	Inferior fronto-occipital fasciculus	R	28	34	0	0.003	150
	Inferior fronto-occipital fasciculus	L	−27	32	9	0.004	174
	Cingulum	R	9	2	33	0.007	37
	Cingulum	L	−8	3	31	0.009	34
Projection fiber	Anterior corona radiation	R	17	28	20	0.002	877
	Anterior corona radiation	L	−24	29	7	0.003	1037
	Superior corona radiation	R	17	15	30	0.003	276
	Superior corona radiation	L	−17	−6	36	0.004	275
	Posterior corona radiation	R	22	−30	34	0.007	106
	Posterior corona radiation	L	−19	−30	35	0.009	25
	Anterior limb of internal capsule	R	22	21	7	0.007	45
	Anterior limb of internal capsule	L	−21	20	8	0.005	78
	Precentral gyrus	L	−19	−12	43	0.007	149
	External capsule	R	28	12	17	0.007	25
	External capsule	L	−26	17	8	0.005	24

Abbreviation. MNI: Montreal Neurological Institute; WM: white matter; R: right; L: left.

Note. Coordinates for the peak voxels are displayed.

### VOI results

The 22 brain regions showing significantly reduced FA in the IAD group were extracted for VOI-based analysis of other diffusion indices. The results are listed in Table 3. Seventeen out of the 22 VOIs showed significantly increased Dr (*p*<0.05, Bonferroni correction for 22 comparisons). No significant differences were detected in Da in any of the VOIs.

**Table 3:** Group differences in diffusivity indices from volume-of-interests (corrected for age).

Anatomic region	Da (×10^−3^ mm^2^/s) (Mean±SD)			Dr (×10^−3^ mm^2^/s) (Mean±SD)			MD (×10^−3^ mm^2^/s) (Mean±SD)		
	CON	IAD	F value	CON	IAD	F value	CON	IAD	F value
Orbital frontal WM R	1.12±0.08	1.17±0.08	3.24	0.66±0.08	0.74±0.06	11.93*	0.81±0.08	0.89±0.07	8.89
Orbital frontal WM L	1.36±0.09	1.33±0.07	0.66	0.58±0.04	0.62±0.04	7.62	0.84±0.05	0.86±0.04	1.23
Genu of corpus callosum R	1.51±0.08	1.56±0.11	1.79	0.45±0.04	0.53±0.05	29.33*	0.80±0.05	0.88±0.06	14.19*
Genu of corpus callosum L	1.58±0.08	1.64±0.10	2.66	0.42±0.04	0.49±0.04	24.04*	0.81±0.04	0.87±0.05	15.08*
Body of corpus callosum R	1.37±0.07	1.43±0.09	4.13	0.48±0.05	0.57±0.05	28.47*	0.78±0.04	0.86±0.05	22.83*
Body of corpus callosum L	1.37±0.09	1.38±0.06	0.29	0.48±0.04	0.55±0.05	21.26*	0.78±0.04	0.83±0.04	15.94*
Splenium R	1.51±0.07	1.51±0.06	0.003	0.40±0.06	0.44±0.03	6.99	0.77±0.06	0.80±0.03	3.19
Inferior fronto-occipital fasciculus R	1.17±0.04	1.16±0.06	0.46	0.61±0.04	0.66±0.03	15.83*	0.79±0.04	0.83±0.04	5.06
Inferior fronto-occipital fasciculus L	1.15±0.05	1.16±0.05	0.21	0.58±0.03	0.63±0.03	16.82*	0.77±0.03	0.80±0.04	7.63
Cingulum R	1.27±0.12	1.32±0.10	1.48	0.46±0.07	0.55±0.06	18.30*	0.73±0.07	0.80±0.05	14.91*
Cingulum L	1.27±0.11	1.30±0.14	0.40	0.44±0.06	0.53±0.05	20.24*	0.72±0.06	0.79±0.06	10.68
Anterior corona radiation R	1.31±0.04	1.31±0.08	0.04	0.55±0.03	0.61±0.04	31.42*	0.80±0.03	0.84±0.04	11.02
Anterior corona radiation L	1.27±0.05	1.26±0.05	0.19	0.55±0.04	0.60±0.03	18.53*	0.79±0.03	0.82±0.03	7.57
Superior corona radiation R	1.23±0.04	1.22±0.05	0.01	0.51±0.02	0.56±0.03	37.68*	0.75±0.02	0.78±0.02	17.12*
Superior corona radiation L	1.25±0.05	1.23±0.05	1.02	0.50±0.03	0.55±0.03	20.65*	0.75±0.03	0.77±0.02	9.89
Posterior corona radiation R	1.19±0.04	1.16±0.05	2.53	0.58±0.04	0.61±0.03	7.94	0.78±0.03	0.79±0.02	1.66
Posterior corona radiation L	1.22±0.08	1.14±0.07	9.25	0.58±0.04	0.61±0.05	2.41	0.80±0.04	0.78±0.04	0.52
Anterior limb of internal capsule R	1.18±0.08	1.22±0.08	2.78	0.50±0.04	0.58±0.05	24.28*	0.73±0.04	0.79±0.05	17.92*
Anterior limb of internal capsule L	1.23±0.07	1.23±0.08	0.04	0.46±0.05	0.52±0.04	10.64	0.72±0.05	0.76±0.05	5.32
Precentral gyrus L	1.27±0.07	1.24±0.06	1.72	0.46±0.02	0.50±0.03	20.89*	0.73±0.03	0.75±0.03	3.88
External capsule R	1.13±0.06	1.14±0.07	0.24	0.60±0.04	0.67±0.05	16.90*	0.78±0.03	0.83±0.05	10.13
External capsule L	1.23±0.05	1.28±0.09	4.05	0.44±0.04	0.50±0.04	22.17*	0.70±0.03	0.76±0.04	26.65*

*p<0.05/22≈0.002 (ANCOVA with age as a covariate variable, Bonferroni corrected for multiple comparisons).

Abbreviation. WM: white matter; CON: controls; IAD: Internet addiction disorder; Da: axial diffusivity; Dr: radial diffusivity; MD: mean diffusivity; R: right; L: left. SD: standard deviation.

**Figure 2:**
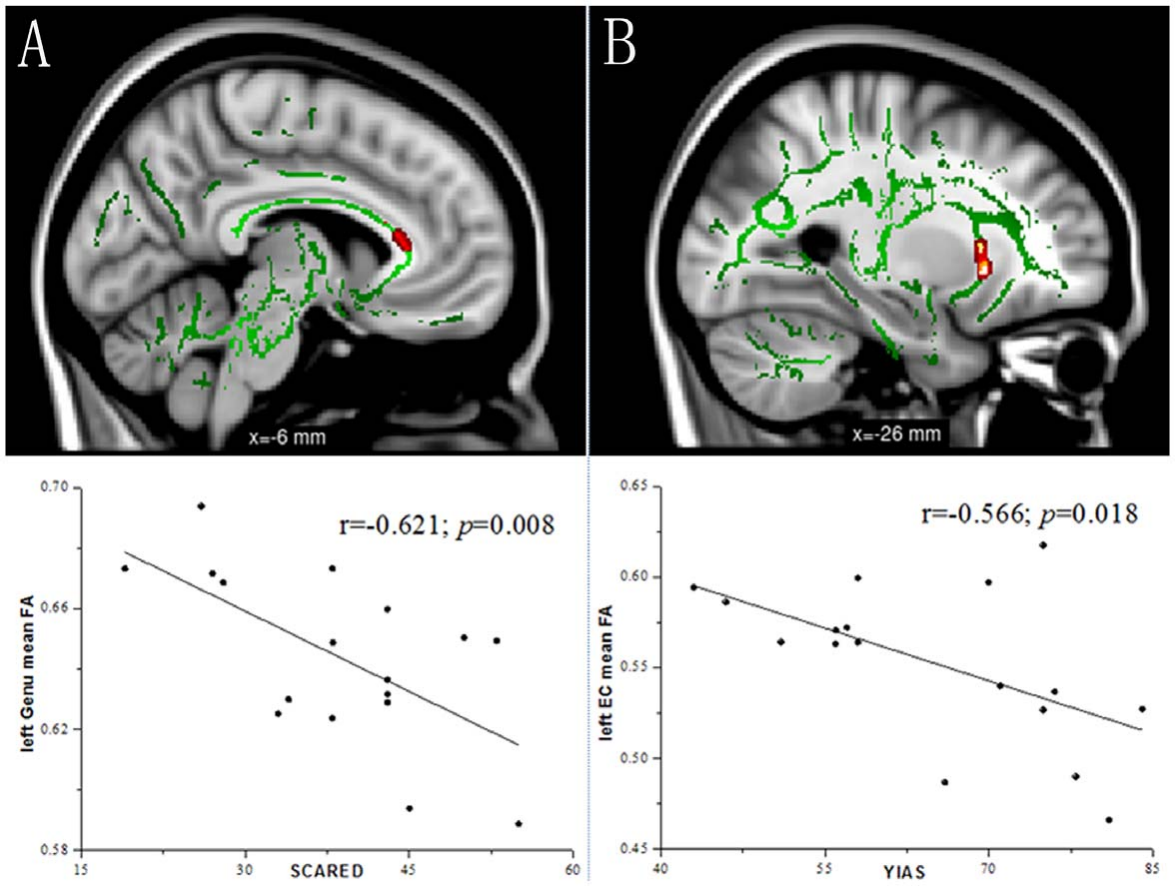
Correlation analysis between fractional anisotropy (FA) and behavioral measures within the Internet addiction disorder (IAD) group. To aid visualization, regions showing significant correlations (red) are thickened using the tbss_fill script implemented in FSL. Figure 2A shows FA values in the left genu of the corpus callosum correlates negatively with the Screen for Child Anxiety Related Emotional Disorders (SCARED) (r = −0.621, *p* = 0.008). Figure 2B shows FA values in the left external capsule correlates negatively with the Young's Internet addiction scale (YIAS) (r = −0.566, *p* = 0.018).

For the 22 VOIs, Pearson correlation analysis demonstrated significantly negative correlations between FA values in the left genu of the corpus callosum and SCARED (r = −0.621, *p* = 0.008, uncorrected; Figure 2A), and between FA values in the left external capsule and YIAS (r = −0.566, *p* = 0.018, uncorrected; Figure 2B) in the IAD subjects. Multiple linear regression analysis showed that the effects of SCARED on the FA within the left genu of the corpus callosum was statistically significant (standardized β = −0.621, t = −3.07, *p* = 0.008), but not that of age, gender, education and other psychometric variables. Multiple linear regression analysis also demonstrated that the effects of YIAS on the FA within the left external capsule was statistically significant (standardized β = −0.566, t = −2.66, *p* = 0.018), but not that of age, gender, education and other psychometric variables.

## Discussion

In this study, we used DTI to investigate the integrity of white matter in IAD adolescents by the observer-independent whole brain voxel-wise TBSS analysis. Compared with the age, gender and education matched controls, IAD subjects had significantly reduced FA in the orbito-frontal white matter, together with cingulum, commissural fibers of the corpus callosum, association fibers including the inferior front-occipital fasciculus, and projection fibers comprising the corona radiation, internal capsule and external capsule (Figure 1 and Table 2). These results provide evidences of widespread deficits in white matter integrity and reflect a disruption in the organization of white matter tracts in IAD. VOI analysis showed that decreased FA observed in IAD was mainly a result of increased radial diffusivity (Table 3), perhaps a manifestation of demyelination. Furthermore, the results of correlation analyses showed FA in the left genu of the corpus callosum was negatively correlated with SCARED, and FA in the left external capsule was negatively correlated with YIAS (Figure 2). These findings suggest that white matter integrity may serve as a potential new treatment target for IAD, and FA may be used as a qualified biomarker to understand the underlying neural mechanisms of injury or to assess the effectiveness of specific early interventions in IAD.

### Abnormal white matter integrity in IAD

The orbito-frontal cortex has extensive connections with prefrontal, visceromotor, and limbic regions, as well as the association areas of each sensory modality [33]. It plays a critical role in emotional processing and addiction-related phenomena, such as craving, compulsive-repetitive behaviors, and maladaptive decision-making [34], [35]. Previous studies found that abnormal white matter integrity in the orbito-frontal cortex has been frequently observed in the subjects exposed to addictive substances, such as alcohol [36], cocaine [37], [38], marijuana [39], methamphetamine [40], and ketamine [41]. Our finding that IAD is associated with impaired white matter integrity in the orbito-frontal regions is consistent with these previous results.

Anterior cingulate cortex (ACC) connects to the frontal lobes and the limbic system, playing an essential role in cognitive control, emotional processing and craving [42]. Abnormal white matter integrity in the anterior cingulum has also been consistently observed in other forms of addiction, such as alcoholism [36], heroin dependence [43], and cocaine addiction [38]. The observation of decreased FA within the anterior cingulum of IAD subjects is consistent with these previous results and with the report that heavy internet overuse [17] is associated with impaired cognitive control. More interestingly, the same group of IAD subjects had been shown to have significantly decreased gray matter density in the left ACC, compared to control [12]. Similar results have also been reported by another group [13].

Another major structure showing reduced FA in IAD subject is the corpus callosum, which is the largest white matter fiber tract connecting neocortex of the two hemispheres [44]. The anterior parts of corpus callosum connect the frontal cortices, while the body and splenium connect parietal, temporal, and occipital homotopic regions [45]. Compromised fiber connectivity within the corpus callosum is a common finding in subjects with substance dependence [46]. In cocaine-dependent subjects, significantly reduced FA in the genu and rostral body [47] and the body and splenium of the corpus callosum [48] were reported. Methamphetamine abusers showed reduced white matter integrity in the genu [49] and rostral body [50] of the corpus callosum. Alcoholism is also associated with decreased FA in the genu, body and splenium of the corpus callosum [51], [52]. Most recently, Bora et al. [53] observed FA reductions in the genu and isthmus of the corpus callosum in opiate-dependent patients. Our findings of reduced FA mainly in the bilateral genu and body of the corpus callosum in IAD subjects suggest that heavy internet overuse, similar to substance abuse, may damage white matter microstructure of the corpus callosum.

Compared to controls, IAD subjects also showed significantly decreased FA in the anterior limb of the internal capsule, external capsule, corona radiation, inferior fronto-occipital fasciculus and precentral gyrus. Again, similar white matter abnormalities had also been observed in other forms of addiction. For example, white matter alterations in the anterior limb of the internal capsule and external capsule have been reported in alcohol abuse [54], [55] and opiate addiction [53]. FA decreases in the anterior limb of the internal capsule may be indicative of alterations in frontal-subcortical circuits. This pathway provides connections between the thalamus/striatum and frontal cortical regions and comprises a system that plays a role in reward and emotional processing [56]. External capsule connects the ventral and medial prefrontal cortex to the striatum. The corona radiata is comprised of white matter fibers linking the cerebral cortex to the internal capsule and provide important connections between the frontal, parietal, temporal, and occipital lobes [57]. Abnormal white matter integrity in corona radiata has been previously observed in cocaine [58] and methamphetamine abuse [59], and alcohol dependence [54]. The inferior fronto-occipital fasciculus is an association bundle connecting the frontal with the parietal and occipital lobes. Compared to the light drinkers, the alcoholics have lower FA in this region [54]. Abnormal precentral gyrus was also reported in heroin dependence [43] and marijuana and alcohol-using adolescents [39].

Overall, our findings indicate that IAD has abnormal white matter integrity in brain regions involving in emotional generation and processing, executive attention, decision making and cognitive control. The results also suggest that IAD may share psychological and neural mechanisms with other types of substance addiction and impulse control disorders.

### Possible mechanisms underlying FA decrease

Although decreased FA is a well-established biomarker for impaired white matter integrity, its exact neurobiological meaning remains to be understood fully. FA of white matter fibers/bundles may be affected by many factors including myelination, axon size and density, path geometry, and extracellular water space between fibers [20]. In this study, we found that the FA reduction in the brain of IAD subjects was mainly driven by an increase in the radial diffusivity, without much changes observed in the axial diffusivity (Table 3). This also appeared to be true in other form of substance dependence, such as cocaine [60], [61], opiate [53], and methamphetamine abuse/addiction [62]. Although it is still a subject of debate, it is generally believed that the radial diffusivity mainly reflects the integrity and thickness of myelin sheets covering the axons [22], while the axial diffusivity may index the organization of the fiber structure and axon integrity [63]. If this assumption holds true in our case, it then may be concluded that reduced FA observed the brain of IAD subjects is most likely a manifestation of disrupted integrity of myelin in the affected brain regions.

### Relationship between FA and behavioral measures in IAD

Behavioral acessment demonstrated that the IAD subjects had significantly higher scores on YIAS, SDQ, SCARED and FAD, compared to control. These findings are consistent with the results of previous neuropsychological studies on IAD subjects [9], [64]. Understanding the associations between white matter integrity and behavioral features provides important insights into the neurobiological mechanisms underlying different aspects of addiction symptoms. For example, Pfefferbaum and colleagues [65] reported a positive correlation between FA values in the splenium and working memory in chronic alcoholics. In cocaine dependce, a significant negative correlation between FA in the anterior corpus callosum and impulsivity, and a positive correlation between FA and discriminability were observed [47]. FA in the right frontal sub-gyral of heroin-dependent subjects was found negatively correlated with the duration of heroin use [43]. Poorer cognitive control was associated with lower FA in the genu of the corpus callosum in methamphetamine abusers [49].

In this study, we investigate the behavioral correlates of FA reduction in the affected brain regions in the IAD subjects. Reduction of FA in the left genu of the corpus callosum of the IAD subjects correlated significantly with increase of SCARED score; while higher YIAS scores appeared to be associated with more severely impaired white matter integrity in the left external capsule.

The SCARED is a reliable and valid self-report questionnaire that measures symptoms of anxiety disorders in children [30]. Neuropsychological studies revealed that IAD adolescents had significantly higher SCARED score than those without IAD [64]. The negative association between SCARED scores and FA in the left genu of the corpus callosum may arise from a disruption connection between the bilateral prefrontal cortices involved in anxiety disorders. The YIAS assesses the degree to which heavy internet usage negatively impact social functioning and relationships [26]; and it is a widely used instrument for evaluating the dependence of the Internet. Previous psychometric studies had demonstrated that IAD subjects had higher YIAS scores than those without IAD [9]. The negative correlation between YIAS scores and FA values in the left external capsule implied that IAD subjects with higher YIAS scores appeared to have lower white matter integrity in the fronto-temporal pathway connected through the external capsule.

In addition, the associations between white matter integrity and behavioral features indicate a novel potential target for treatment of IAD subjects, which is consistent with recent calls to focus on cognitive enhancement among addicted populations including IAD subjects [66], [67]. Recent studies have shown that physical or pharmacological treatments may improve white matter integrity. For example, Schlaug and colleagues reported that physical therapy could enhance white matter integrity in the right language area and improve speech in aphasic patients with lesions in the left language area [68]. Therefore, the findings of significant associations between impaired white matter integrity across extensive regions and poorer neuropshychological measures in IAD subjects suggest that white matter integrity may serve as a predictor of abstinence or a potential new treatment target for IAD.

### TBSS vs. VBM

Our previous study showed that there was no white matter atrophy in the same cohort IAD subjects [12], and this might appear to be inconsistent with the findings in this study. Gray or white matter density measured by VBM is defined as the relative concentration of gray or white matter structures in spatially normalized images (i.e. the proportion of gray or white matter to all tissue types within a region), which should not “be confused with cell packing density measured cytoarchitectonically” [69]. In the DTI/TBSS analysis, FA value is used as a surrogate of structural integrity of white matter, which may come about through factors such as myelination, axon size and density, path geometry, and extracellular water space between fibers [20]. Therefore, VBM-derived density and structural integrity measured by DTI represent different aspects of white matter. There can be white matter regions showing no atrophy by VBM, but structurally impaired as detected by FA measurements (i.e., it is exactly the case in our study of IAD), and vice versa. Taking the findings from the two studies together, it may be concluded that IAD in adolescence is not associated with morphological changes in white matter at the macroscopic level, but rather impaired white matter microstructural integrity, which might be attributed to demyelination.

### Limitations of the Study

There are several limitations that should be mentioned in this study. Firstly, the diagnosis of IAD was mainly based on results of self-reported questionnaires, which might cause some error classification. Therefore, the diagnosis of IAD needs to be refined with standardized diagnostic tools to improve the reliability and validity. Secondly, although we tried our best to exclude comorbid substance and psychiatric disorders, it is acknowledged that this may not have been done sufficiently (i.e., no urine test was given, sleep habits and schedules and daily sleepiness were not controlled in the experiment design), such that the white matter changes observed may not be attributed to IAD per se. It is also admitted that this is not a controlled study of effects of internet use on brain structure. Thirdly, the sample size in this study was relatively small, which might reduce the power of the statistical significance and generalization of the findings. Owing to this limitation, these results should to be considered preliminary, which need to be replicated in future studies with a larger sample size. Lastly, as a cross-sectional study, our results do not clearly demonstrate whether the psychological features preceded the development of IAD or were a consequence of the overuse of the Internet. Therefore, future studies should attempt to identify the causal relations between IAD and the psychological measures.

In conclusion, we used DTI with TBSS analysis to investigate the microstructure of white matter among IAD adolescents. The results demonstrate that IAD is characterized by impairment of white matter fibers connecting brain regions involved emotional generation and processing, executive attention, decision making, and cognitive control. The findings also suggest that IAD may share psychological and neural mechanisms with other types of impulse control disorders and substance addiction. In addition, the associations between FA values in white matter regions and behavioral measures indicate that white matter integrity may serve as a potential new treatment target for IAD, and DTI may be valuable in providing information on prognosis for IAD, and FA may be a qualified biomarker to assess the effectiveness of specific early interventions in IAD.

## References

[1] Aboujaoude E (2010). Problematic Internet use: an overview.. World Psychiatry.

[2] Beard KW, Wolf EM (2001). Modification in the proposed diagnostic criteria for Internet addiction.. Cyberpsychol Behav.

[3] Young KS (1998). Internet addiction: the emergence of a new clinical disorder.. Cyberpsychol Behav.

[4] Chou C, Condron L, Belland JC (2005). A review of the research on internet addiction.. Educ Psychol Rev.

[5] Douglas AC, Mills JE, Niang M, Stepchenkova S, Byun S (2008). Internet addiction: Meta-synthesis of qualitative research for the decade 1996–2006.. Comput Human Behav.

[6] Weinstein A, Lejoyeux M (2010). Internet addiction or excessive internet use.. Am J Drug Alcohol Abuse.

[7] Bernardi S, Pallanti S (2009). Internet addiction: a descriptive clinical study focusing on comorbidities and dissociative symptoms.. Compr Psychiatry.

[8] Caplan SE (2002). Problematic Internet use and psychosocial well-being: Development of a theory-based cognitive-behavioral measurement instrument.. Comput Human Behav.

[9] Cao F, Su L (2007). Internet addiction among Chinese adolescents: prevalence and psychological features.. Child Care Health Dev.

[10] Shaw M, Black DW (2008). Internet addiction: definition, assessment, epidemiology and clinical management.. CNS Drugs.

[11] Tao R, Huang XQ, Wang JN, Zhang HM, Zhang Y (2010). Proposed diagnostic criteria for internet addiction.. Addiction.

[12] Zhou Y, Lin FC, Du YS, Qin LD, Zhao ZM (2011). Gray matter abnormalities in Internet addiction: A voxel-based morphometry study.. Eur J Radiol.

[13] Yuan K, Qin W, Wang G, Zeng F, Zhao L (2011). Microstructure abnormalities in adolescents with internet addiction disorder.. PLoS One.

[14] Liu J, Gao XP, Osunde I, Li X, Zhou SK (2010). Increased regional homogeneity in internet addiction disorder: a resting state functional magnetic resonance imaging study.. Chin Med J (Engl).

[15] Han DH, Bolo N, Daniels MA, Arenella L, Lyoo IK (2011). Brain activity and desire for Internet video game play.. Compr Psychiatry.

[16] Ko CH, Liu GC, Hsiao S, Yen JY, Yang MJ (2009). Brain activities associated with gaming urge of online gaming addiction.. J Psychiatr Res.

[17] Dong G, Lu Q, Zhou H, Zhao X (2010). Impulse inhibition in people with internet addiction disorder: electrophysiological evidence from a Go/NoGo study.. Neurosci Lett.

[18] Park HS, Kim SH, Bang SA, Yoon EJ, Cho SS (2010). Altered regional cerebral glucose metabolism in internet game over users: a 18F-fluorodeoxyglucose positron emission tomography study.. CNS Spectr.

[19] Basser PJ, Mattiello J, LeBihan D (1994). Estimation of the effective self-diffusion tensor from the NMR spin echo.. J Magn Reson B.

[20] Le Bihan D (2003). Looking into the functional architecture of the brain with diffusion MRI.. Nat Rev Neurosci.

[21] Basser PJ, Pierpaoli C (1996). Microstructural and physiological features of tissues elucidated by quantitative diffusion tensor MRI.. J Magn Reson B.

[22] Song SK, Sun SW, Ramsbottom MJ, Chang C, Russell J (2002). Dysmyelination revealed through MRI as increased radial (but unchanged axial) diffusion of water.. Neuroimage.

[23] Smith SM, Jenkinson M, Johansen-Berg H, Rueckert D, Nichols TE (2006). Tract-based spatial statistics: voxelwise analysis of multi-subject diffusion data.. Neuroimage.

[24] Oldfield RC (1971). The assessment and analysis of handedness: the Edinburgh inventory.. Neuropsychologia.

[25] Sheehan DV, Sheehan KH, Shytle RD, Janavs J, Bannon Y (2010). Reliability and validity of the Mini International Neuropsychiatric Interview for Children and Adolescents (MINI-KID).. J Clin Psychiatry.

[26] Young KS (1998). Caught in the Net: How to recognize the signs of Internet addiction and a winning strategy for recovery.

[27] Huang X, Zhang Z (2001). The compiling of the adolescence time management disposition scale.. Acta Psychol Sin.

[28] Goodman R (1997). The Strengths and Difficulties Questionnaire: a research note.. J Child Psychol Psychiatry.

[29] Patton JH, Stanford MS, Barratt ES (1995). Factor structure of the Barratt impulsiveness scale.. J Clin Psychol.

[30] Birmaher B, Khetarpal S, Brent D, Cully M, Balach L (1997). The Screen for Child Anxiety Related Emotional Disorders (SCARED): scale construction and psychometric characteristics.. J Am Acad Child Adolesc Psychiatry.

[31] Epstein NB, Baldwin LM, Bishop DS (1983). The McMaster family assessment device.. J Marital Fam Ther.

[32] Smith SM (2009). Threshold-free cluster enhancement: addressing problems of smoothing, threshold dependence and localization in cluster inference.. Neuroimage.

[33] Ongur D, Price JL (2000). The organization of networks within the orbital and medial prefrontal cortex of rats, monkeys and humans.. Cereb Cortex.

[34] Schoenebaum G, Roesch MR, Stalnaker TA (2006). Orbitofrontal cortex, decision making and drug addiction.. Trends Neurosci.

[35] Volkow ND, Fowler JS (2000). Addiction, a disease of compulsion and drive: Involvement of the orbitofrontal cortex.. Cereb Cortex.

[36] Harris GJ, Jaffin SK, Hodge SM, Kennedy D, Caviness VS (2008). Frontal white matter and cingulum diffusion tensor imaging deficits in alcoholism.. Alcohol Clin Exp Res.

[37] Lim KO, Choi SJ, Pomara N, Wolkin A, Rotrosen JP (2002). Reduced frontal white matter integrity in cocaine dependence: A controlled diffusion tensor imaging study.. Biol Psychiatry.

[38] Romero MJ, Asensio S, Palau C, Sanchez A, Romero FJ (2010). Cocaine addiction: diffusion tensor imaging study of the inferior frontal and anterior cingulate white matter.. Psychiatry Res.

[39] Bava S, Frank LR, McQueeny T, Schweinsburg BC, Schweinsburg AD (2009). Altered white matter microstructure in adolescent substance users.. Psychiatry Res.

[40] Alicata D, Chang L, Cloak C, Abe K, Ernst T (2009). Higher diffusion in striatum and lower fractional anisotropy in white matter of methamphetamine users.. Psychiatry Res.

[41] Liao Y, Tang J, Ma M, Wu Z, Yang M (2010). Frontal white matter abnormalities following chronic ketamine use: a diffusion tensor imaging study.. Brain.

[42] Goldstein RZ, Volkow ND (2002). Drug addiction and its underlying neurobiological basis: neuroimaging evidence for the involvement of the frontal cortex.. Am J Psychiatry.

[43] Liu H, Li L, Hao Y, Cao D, Xu L (2008). Disrupted white matter integrity in heroin dependence: a controlled study utilizing diffusion tensor imaging.. Am J Drug Alcohol Abuse.

[44] deLacoste MC, Kirkpatrick JB, Ross ED (1985). Topography of the human corpus callosum.. J Neuropathol Exp Neurol.

[45] Abe O, Masutani Y, Aoki S, Yamasue H, Yamada H (2004). Topography of the human corpus callosum using diffusion tensor tractography.. J Comput Assist Tomogr.

[46] Arnone D, Abou-Saleh MT, Barrick TR (2006). Diffusion tensor imaging of the corpus callosum in addiction.. Neuropsychobiology.

[47] Moeller FG, Hasan KM, Steinberg JL, Kramer LA, Dougherty DM (2005). Reduced anterior corpus callosum white matter integrity is related to increased impulsivity and reduced discriminability in cocaine-dependent subjects: diffusion tensor imaging.. Neuropsychopharmacology.

[48] Lim KO, Wozniak JR, Mueller BA, Franc DT, Specker SM (2008). Brain macrostructural and microstructural abnormalities in cocaine dependence.. Drug Alcohol Depend.

[49] Salo R, Nordahl TE, Buonocore MH, Natsuaki Y, Waters C (2009). Cognitive control and white matter callosal microstructure in methamphetamine-dependent subjects: a diffusion tensor imaging study.. Biol Psychiatry.

[50] Moeller FG, Steinberg JL, Lane SD, Buzby M, Swann AC (2007). Diffusion tensor imaging in MDMA users and controls: association with decision making.. Am J Drug Alcohol Abuse.

[51] De Bellis MD, Van Voorhees E, Hooper SR, Gibler N, Nelson L (2008). Diffusion tensor measures of the corpus callosum in adolescents with adolescent onset alcohol use disorders.. Alcohol Clin Exp Res.

[52] Pfefferbaum A, Adalsteinsson E, Sullivan EV (2006). Dysmorphology and microstructural degradation of the corpus callosum: Interaction of age and alcoholism.. Neurobiol Aging.

[53] Bora E, Yucel M, Fornito A, Pantelis C, Harrison BJ (2010). White matter microstructure in opiate addiction.. Addict Biol.

[54] Yeh PH, Simpson K, Durazzo TC, Gazdzinski S, Meyerhoff DJ (2009). Tract-based spatial statistics (TBSS) of diffusion tensor imaging data in alcohol dependence: Abnormalities of the motivational neurocircuitry.. Psychiatry Res.

[55] Pfefferbaum A, Rosenbloom M, Rohlfing T, Sullivan EV (2009). Degradation of association and projection white matter systems in alcoholism detected with quantitative fiber tracking.. Biol Psychiatry.

[56] Mori S, Wakana S, Nagae-Poetscher L, Van Zijl P (2005). MRI Atlas of Human White Matter.

[57] Wakana S (2004). Fiber tract-based atlas of human white matter anatomy.. Radiology.

[58] Bell RP, Foxe JJ, Nierenberg J, Hoptman MJ, Garavan H (2011). Assessing white matter integrity as a function of abstinence duration in former cocaine-dependent individuals.. Drug Alcohol Depend.

[59] Tobias MC, O'Neill J, Hudkins M, Bartzokis G, Dean AC (2010). White-matter abnormalities in brain during early abstinence from methamphetamine abuse.. Psychopharmacology.

[60] Lane SD, Steinberg JL, Ma LS, Hasan KM, Kramer LA (2010). Diffusion tensor imaging and decision making in cocaine dependence.. PLoS One.

[61] Moeller FG, Hasan KM, Steinberg JL, Kramer LA, Valdes I (2007). Diffusion tensor imaging eigenvalues: Preliminary evidence for altered myelin in cocaine dependence.. Psychiatry Res.

[62] Kim IS, Kim YT, Song HJ, Lee JJ, Kwon DH (2009). Reduced corpus callosum white matter microstructural integrity revealed by diffusion tensor eigenvalues in abstinent methamphetamine addicts.. Neurotoxicology.

[63] Song SK, Sun SW, Ju WK, Lin SJ, Cross AH (2003). Diffusion tensor imaging detects and differentiates axon and myelin degeneration in mouse optic nerve after retinal ischemia.. Neuroimage.

[64] Huang X, Zhang H, Li M, Wang J, Zhang Y (2010). Mental health, personality, and parental rearing styles of adolescents with Internet addiction disorder.. Cyberpsychol Behav Soc Netw.

[65] Pfefferbaum A, Sullivan EV, Hedehus M, Adalsteinsson E, Lim KO (2000). In vivo detection and functional correlates of white matter microstructural disruption in chronic alcoholism.. Alcohol Clin Exp Res.

[66] Du YS, Jiang W, Vance A (2010). Longer term effect of randomized, controlled group cognitive behavioural therapy for Internet addiction in adolescent students in Shanghai.. Aust N Z J Psychiatry.

[67] Vocci FJ (2008). Cognitive remediation in the treatment of stimulant abuse disorders: a research agenda.. Exp Clin Psychopharmacol.

[68] Schlaug G, Marchina S, Norton A (2009). Evidence for plasticity in white-matter tracts of patients with chronic Broca's aphasia undergoing intense intonation-based speech therapy.. Ann N Y Acad Sci.

[69] Mechelli A, Price CJ, Friston KJ, Ashburner J (2005). Voxel-based morphometry of the human brain: Methods and applications.. Curr Med Imaging Rev.

